# Supplementary material for: A Randomized, Double-Blind, Placebo-Controlled Trial to Evaluate the Effects of Multi-Strain Synbiotic in Patients with Functional Diarrhea and High Fecal Calprotectin Levels: A Pilot Study
Source: Nutrients. 2022 Nov 25;14(23):5017. doi: 10.3390/nu14235017 (PMC9735760; doi:10.3390/nu14235017)
Supplement: Supplementary file 1 [file nutrients-14-05017-s001.zip › nutrients-2035166-supplementary.pdf]

Supplementary Table S1. Requirements for Trial Subjects

Inclusion criteria: All of the following must be satisfied
1) Consent to participate in this study and sign a written informed consent
2) Adults between the ages of 20 and 65 years
3) Have difficult bowel movements (according to Rome IV criteria for functional diarrhea)
4) Positive fecal calprotectin test result (≥ 11.5 mg/kg)
**Exclusion criteria: Any of these**
1) Taking probiotics/prebiotics or antibiotics/anti-inflammatory drugs for more than a week within one month of the first visit
2) Consuming a lot of dietary fiber (recommended food score > 36)
3) Currently have an organic disease of the gastrointestinal system, or within the past 2 years (e.g., Crohn's disease, celiac disease, ulcerative colitis, or pancreatitis)
4) Previous gastrointestinal surgery (gastrectomy, small intestine resection, colectomy, or cholecystectomy) except for appendicitis
5) Lactose-intolerant
6) Any of the following or taking related drugs<br>A. Severe hepatic or renal dysfunction, cardiovascular disease (including uncontrolled hypertension), immune disorder (immunocompromised patients, immunosuppressant users), malignancy<br>B. Red flags signs such as hemorrhagic hemorrhoids, anal fissures, severe weight loss, and dysphagia<br>C. Alcohol addiction, psychogenic disorders, anorexia, or bulimia
7) Taking the following drugs within 3 days of the first visit<br>A. Antispasmodics: Cimetropium, Mebeverine, Pinaverium, Trimebutine, etc.<br>B. Prokinetics: Motilium, Levopride, Itopride, Mosapride, etc.<br>C. Antidepressant: tricyclic antidepressant, selective serotonin reuptake inhibitor<br>D. Antidiarrheal: Loperamide
8) Recently traveled to an area with an epidemic parasitic disease
9) Hypersensitivity to the test food or ingredients contained in the test food
10) Participated in another clinical study within one month of the first visit
11) Pregnant or lactating women, or planning to become pregnant
12) Judged as inappropriate to participate in the research by the researcher
